# Research on weed identification method in rice fields based on UAV remote sensing

**DOI:** 10.3389/fpls.2022.1037760

**Published:** 2022-11-09

**Authors:** Fenghua Yu, Zhongyu Jin, Sien Guo, Zhonghui Guo, Honggang Zhang, Tongyu Xu, Chunling Chen

**Affiliations:** ^1^ College of Information and Electrical Engineering, Shenyang Agricultural University, Shenyang, China; ^2^ Department of Science and Technology, Liaoning Agricultural Information Engineering Technology Research Center, Shenyang, China

**Keywords:** rice weeds, UAV, multispectral imaging, vegetation indices, remote sensing

## Abstract

Rice is the world’s most important food crop and is of great importance to ensure world food security. In the rice cultivation process, weeds are a key factor that affects rice production. Weeds in the field compete with rice for sunlight, water, nutrients, and other resources, thus affecting the quality and yield of rice. The chemical treatment of weeds in rice fields using herbicides suffers from the problem of sloppy herbicide application methods. In most cases, farmers do not consider the distribution of weeds in paddy fields, but use uniform doses for uniform spraying of the whole field. Excessive use of herbicides not only pollutes the environment and causes soil and water pollution, but also leaves residues of herbicides on the crop, affecting the quality of rice. In this study, we created a weed identification index based on UAV multispectral images and constructed the *WDVI*_*NIR*_ vegetation index from the reflectance of three bands, RE, G, and NIR. *WDVI*_*NIR*_ was compared with five traditional vegetation indices, NDVI, LCI, NDRE, and OSAVI, and the results showed that *WDVI*_*NIR*_ was the most effective for weed identification and could clearly distinguish weeds from rice, water cotton, and soil. The weed identification method based on *WDVI*_*NIR*_ was constructed, and the weed index identification results were subjected to small patch removal and clustering processing operations to produce weed identification vector results. The results of the weed identification vector were verified using the confusion matrix accuracy verification method and the results showed that the weed identification accuracy could reach 93.47%, and the Kappa coefficient was 0.859. This study provides a new method for weed identification in rice fields.

## 1 Introduction

China is a large country of rice cultivation, and there are more varieties and classifications of rice in China. Field weeds plague the development of rice production and are a major factor in preventing high and stable rice yields (Feng et al., 2018). According to statistics, weed damage alone in 150 million acres of arable land worldwide causes more than $7 billion in losses each year, accounting for approximately one-third of the total damage caused by diseases, insects, and weeds, and directly causes 125 million tons of grain loss (Liu et al., 2014).Since weeds have a fast growth rate and well-developed root system, they are in an advantageous position to compete with rice for growth resources, thus inhibiting rice growth (Liu et al., 2020). Among them, weeds in paddy fields are diverse, with complex grass phase and a long occurrence period (Duarte et al., 2021; De Simone et al., 2022). By competing with rice for water, fertilizer, light, and space, they change the microecological environment of paddy fields, affect the photosynthesis, nutrition, and reproductive growth of rice, and are intermediate hosts of pests and diseases, aggravating the occurrence of pests and diseases, leading to yield reduction and decline of rice quality, and causing huge losses to rice production (Luo et al., 2020). In the current rice weed management process, chemical weed control is currently the most effective and widely used method of weed control in rice fields, commonly used to spray herbicides uniformly and covering the entire operating area in a disorderly “spot” or “sheet” form (Eppinga et al., 2020; Druskin et al., 2021; Wang et al., 2021). The presence of weeds can lead to excessive spraying of herbicides (Siva Kumar et al., 2020; Su et al., 2022). How to achieve accurate application of weed and reduce the use of agrochemicals is a key issue; the prerequisite to solving this problem is to achieve accurate and rapid detection and identification of weeds (Maes and Steppe, 2019). The rice weeds management process, chemical weed control, is currently the most effective and widely used method of weed control in rice fields, commonly used to spray herbicides uniformly and cover the entire operating area in a disorderly “spot” or “sheet” form. The presence of weeds can lead to overspray of herbicides. How to achieve accurate application of weed and reduce the use of agrochemicals is a key issue, and the prerequisite to solving this problem is to achieve accurate and rapid detection and identification of weeds (Carroll, 2020). Based on low-altitude UAV remote sensing technology, we can carry out accurate monitoring of weeds in rice fields and generate agricultural UAV weed application prescription maps, and carry out UAV precision weeding for rice, (Otsu et al., 2019), which is a new idea to solve the current herbicide overapplication problem. The prerequisite of herbicide precision application is to obtain remote sensing images of rice fields and analyze the weed distribution status in them, get a grid-shaped weed distribution map, and generate an herbicide operation prescription map (Matsunami et al., 2009). The use of UAVs to collect remote sensing images of rice fields and perform weed analysis has been similarly reported around the world.

The use of UAV remote sensing technology has enabled rapid image acquisition and weed mapping in crops such as sunflower, mango, and rice (Jin et al., 2022). While identifying weeds in rice, an important issue is the need to locate weeds against a green vegetation background (Stroppiana et al., 2018). When the technologies of remote sensing data acquisition, stitching and correction are more mature, backward research on the resolution of remote sensing data becomes the main bottleneck of remote sensing development (Tao and Wei, 2022). When parsing remote sensing data, machine learning is widely used for image classification, and weed image recognition models have been developed using deep learning neural networks in an increasing number of literatures (Kawamura et al., 2021). Andrea et al. used convolutional neural networks to distinguish maize plants from weeds in the early growth stage of the crop, and trained the convolutional neural networks using the data set generated in the segmentation stage, and the recognition accuracy reached 97.23% (Punithavathi et al., 2023). Flores et al. used support vector machine model (SVM), neural network (NN), random forest (RF), GoogLeNet and VGG-16 models for recognition detection after collecting image shape, color and texture feature values in a greenhouse environment to simulate field conditions, and finally the recognition accuracy of the VGG-16 model in distinguishing soybean seedlings from corn weeds reached. The accuracy of the VGG-16 model in distinguishing soybean seedlings from corn seedlings was 96.2%, which was the highest among the above five model methods (Hirohiko, 2002; Liu and Yu, 2013; Druskin et al., 2021). Sujaritha designed an automatic image classification system for extracting leaf texture using fuzzy real-time classification counting, which was able to correctly identify sugarcane crops among 9 different weeds, and the accuracy of the system in detecting weeds was 92.9% (Sujaritha et al., 2017). Spectral index can provide an important basis for the identification of rice weeds. Many studies have added spectral index to improve the identification accuracy of rice weeds. Barrero et al. used Neural Networks to detect gramineous weeds 50 days after the emergence of rice field using visible light band and NGRDI index image fusion. The M/MGT index values obtained from the detection results ranged from 80 to 108%. MP values range from 70 to 85% (Barrero and Perdomo, 2018). Stroppiana et al. used spectral information, SAVI and GSAVI spectral indices and unsupervised clustering algorithms to classify weeds in the early stages of the growing season, with an overall accuracy higher than 94% (Stroppiana et al., 2018). Kawamura et al. used a combination of hue-saturation-brightness, canopy height model, spatial texture, color index of vegetation extraction and excess green. A classifier combining simple linear iterative clustering algorithm and random forest algorithm was used to identify weeds in the early growth stage of small rice plants. out-of-bag accuracy is higher than 0.915 (Kawamura et al., 2021).

Currently, related research mainly focuses on the identification and detection of weeds in paddy fields, while relatively little research has been conducted on how to generate accurate operation prescription maps for agricultural drones through weed distribution information in paddy fields (Mohidem et al., 2021). Northern coldland rice is usually weeded 15-20 days after transplanting, therefore, in this study, remote sensing images of rice tillering stage were selected to identify weeds. By observing the UAV remote sensing images, weeds in northern cold rice were found to have less differences in textural characteristics, similar shapes and the same color as rice at the tillering stage (Motavalli et al., 2012; Souri et al., 2022). Weeds have group aggregation, and it is difficult to distinguish them from rice using UAV visible remote sensing images, while spectra can reflect their physicochemical information and highlight their aggregation characteristics. Therefore, this paper uses spectra to identify weeds in rice fields. In this study, the DJI Phantom 4 UAV and its multispectral camera were used to collect multispectral remote sensing images of paddy fields (Zhu et al., 2020). With rice weeds as the identification target, the vegetation index was constructed to highlight the spectral characteristics of weeds (Lu and Zhang, 2020; Nawaz et al., 2021). The density partitioning algorithm is used to obtain the distribution information of the weeds in the rice field and generate the weed distribution map with the best classification effect (Wang et al., 2019). It provides a decision basis for the application of precision pesticides by agriculture UAV.

## 2 Materials and methods

### 2.1 Study area and experimental details

The trial site was located at the precision agriculture aerial research base of Shenyang Agricultural University, Gengzhuang Town, Haicheng City, Liaoning Province (40° 58’ 45.39” N, 122° 43’ 47.01” E), and the test variety was “Japonica 653”, a variety widely grown in Liaoning. In this study, the UAV multispectral images and visible images were collected separately from the test field on June 23, 2021. The weeds in the study area were mainly barnyard grass and Monochoria korsakowii Regel & Maack, which were verified in the field.

### 2.2 Data acquisition

The multispectral remote sensing image data collection equipment was Phantom 4 RTK UAV combined with ground station software DJI GS PRO for route planning. Multispectral remote sensing UAV flight altitude of 25 meters, UAV longitudinal and lateral route overlap rate of 85%. six 1/2.9-inch CMOS, including five monochrome sensors for multispectral imaging single sensor, effective pixels 2.08 million. five characteristic wavelength specific information as shown in **Table 1**.

**Table 1:** Characteristic wavelengths of multispectral UAV remote sensing platform.

Name	Central wavelength	Wavelength range
Blue (B)	450 nm	± 16 nm
Green (G)	560 nm	± 16 nm
Red (R)	650 nm	± 16 nm
Red edge (RE)	730 nm	± 16 nm
Near Infrared (NIR)	840 nm	± 26 nm

The multispectral camera has an FOV of 62.7°, a focal length of 5.74 mm, and an aperture of f/2.2. Monochrome sensors gain in the range of 1-8 multiples.The flying speed of the UAV is set to 5m/s, the altitude is 30m, and the heading and side-direction repetition rate is 80%.

The Phantom 4 RTK quadrotor UAV was used as the flight platform to acquire visible light remote sensing images, with a built-in RTK differential positioning system and a positioning accuracy of 1 cm + 1 ppm, 1 ppm means that the error increases by 1 mm for every 1 km of flight (Lambert et al., 2019; Niu et al., 2021). DJI flight software was used to plan the route of the test area, and orthophoto raw data from the test field were obtained by taking photos at regular intervals.

In this study, multispectral and visible images were acquired for weed identification using a Phantom 4 RTK UAV on June 18, 2021 (Wei et al., 2021). The validation data in this study were visually interpreted using a manual visual interpretation method for the visible images, and a total of 141,483 pixel points were selected, including 48,255 pixel points for the weed category and 93,228 pixel points for the non-weed category.

### 2.3 UAV remote sensing image processing

Pix4D image processing software was used to orthorectify and crop the visible images of the test area collected by UAV, and finally high-resolution orthophotos of the rice fields were obtained.

When the Phantom 4 RTK multispectral UAV remote sensing platform observes the target radiant energy, the radiation distortion caused by the sensor response characteristics and external natural conditions (including solar radiation conditions and atmospheric transmission conditions, etc.) causes distortion of the remote sensing images and affects the interpretation and decoding of remote sensing images; therefore, the radiation calibration of multispectral images is needed. In this study, first, three reflectivity plates with 60% reflectivity were laid flat on the ground near the measurement area, and the Phantom 4 RTK multispectral took off to a height of 7 times the side length of the plates, adjusted the aircraft position so that the plates were in the center of the camera frame and ensured that there was no shadow on the plates, then adjusted the gimbal to -90°, kept the EV value at 0 and took a set of photos manually (Naji, 2018). The multispectral image is used to correct the reflectivity of the acquired UAV remote sensing image.

### 2.4 Research methods

#### 2.4.1 Construction of Vegetation Index

Most of the existing multispectral remote sensing UAV images are used as input of the weed identification model by the NDVI, EVI, DVI and other indices, but the above vegetation indices are more used to carry out inversion studies of physical and chemical parameters of rice, while the accuracy of rice weed identification still has some shortcomings (Clevers and Verhoef, 1993). In this study the characteristic vegetation indices of the weed(WDVI) were constructed by analyzing the spectral characteristics between the weeds and the rice, and the specific construction methods are as follows.

(1) UAV multispectral wavelengths of *x*_B_、*x*_G_、*x*_R_、*x*_RE_、*x*_NIR_ .
(2) Selection of the band *x*_*t*_(*t*∈B、G……*NIR*) as the characteristic transfer band.
(3) Construct the characteristic spectral ratio of multiple groups using other characteristic bands *x*_*f*_(*f*∈B、G……*NIR*, and *f*≠*t*) as a ratio to *x*_*t*_ , both $W_{f}=\frac{x_{f}}{x_{t}}$ .
(4) After taking the logarithm of the ratio result, the correlation with nitrogen content remained good. Therefore, two sets of characteristic spectral ratios *W*_*f*_(*f*∈B、G……*NIR*) , were selected and the characteristic transfer index of weeds(WDVI) was constructed using Equation 1:

(1) $$WDVI={log}_{W_{f}}B_{f}={log}_{\frac{x_{b}}{x_{t}}}\frac{x_{a}}{x_{t}}$$

In this study, five vegetation indices were constructed using five bands, as shown in **Table 2**

**Table 2:** Five medium Combination Vegetation Index.

Name	Formula
WDVI1	$WDVI_{NIR}=\log_{\frac{\text{G}}{\text{NIR}}}\frac{RE}{NIR}$
WDVI2	$WDVI_{NIR}=\log_{\frac{\text{R}}{\text{NIR}}}\frac{RE}{NIR}$
WDVI3	$WDVI_{NIR}=\log_{\frac{\text{RE}}{\text{NIR}}}\frac{R}{NIR}$
WDVI4	$WDVI_{NIR}=\log_{\frac{\text{R}}{\text{NIR}}}\frac{G}{NIR}$
WDVI5	$WDVI_{NIR}=\log_{\frac{\text{G}}{\text{NIR}}}\frac{R}{NIR}$

#### 2.4.2 Weed identification modeling methods

Threshold segmentation is the earliest method studied and used in image segmentation, which has the characteristics of clear physical meaning, easy implementation, and good real-time performance (Setojima et al., 1989; Qin et al., 2013). According to the regional weed distribution map after visual interpretation of visible light remote sensing images and experience knowledge, this study adjusts the gray segmentation threshold of the multispectral index to determine the distribution range of weeds in the index. The grid threshold partition mapping function is as follows:


(2) $$f(x,y)=\{\begin{array}{cc} 0 & 0\leq f(x,y)\leq t\\ L-1 & t<(x,y)\leq L-1 \end{array}$$

Let the size of the raster image be M × N, and the gray level number be *L*, and f (*x, y*) denotes the gray level of the pixel with coordinates (*, y*), where x ∈ [1, M] and y ∈ [1, N].

According to the gray segmentation threshold, the grid image of weed distribution is extracted (Bouman et al., 1992). The algorithm to remove small patches is used to remove scattered grids in the grid images, and the spatial distribution map of weeds is obtained. The grid resampling algorithm was used to resample the grid to 1m × 1m, and the UAV application prescription diagram was generated. Weed analysis process as shown in **Figure 2**.

**Figure 1:**
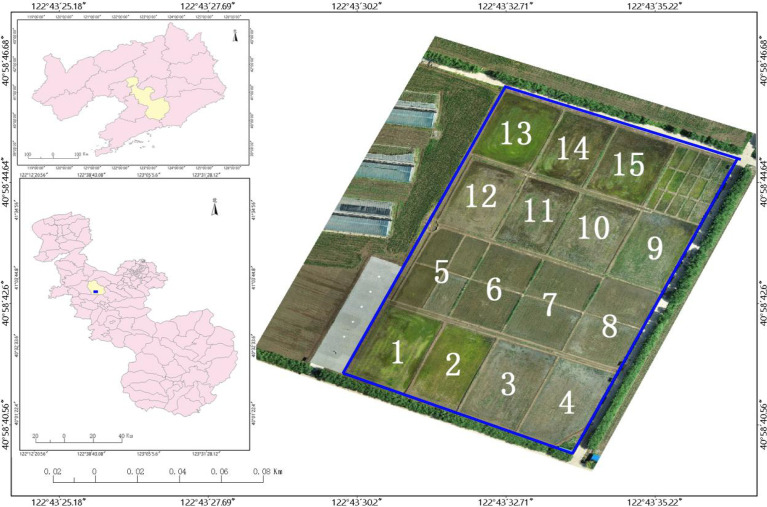
Location map of test site.

**Figure 2:**
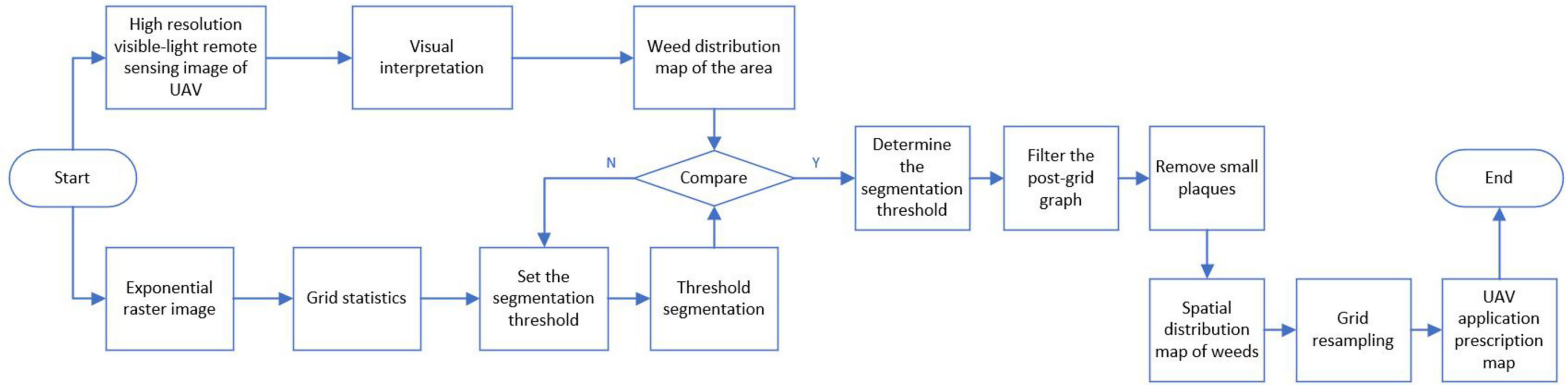
Rice weed identification process.

### 2.5 Evaluation indicators

Confusion matrix is a standard format for representing accuracy evaluation in the form of a matrix with n rows and n columns. In image accuracy evaluation, it is mainly used to compare the classification results with the actual measured values, and the accuracy of the classification results can be displayed inside a confusion matrix. The confusion matrix is calculated by comparing the position and classification of each actual measured image element with the corresponding position and classification in the classified image. In this study, the overall accuracy of the confusion matrix and the Kappa coefficient are used as classification effectiveness evaluation metrics.

## 3 Results and analysis

### 3.1 Results of vegetation index for weed identification in rice

**Figure 3:**
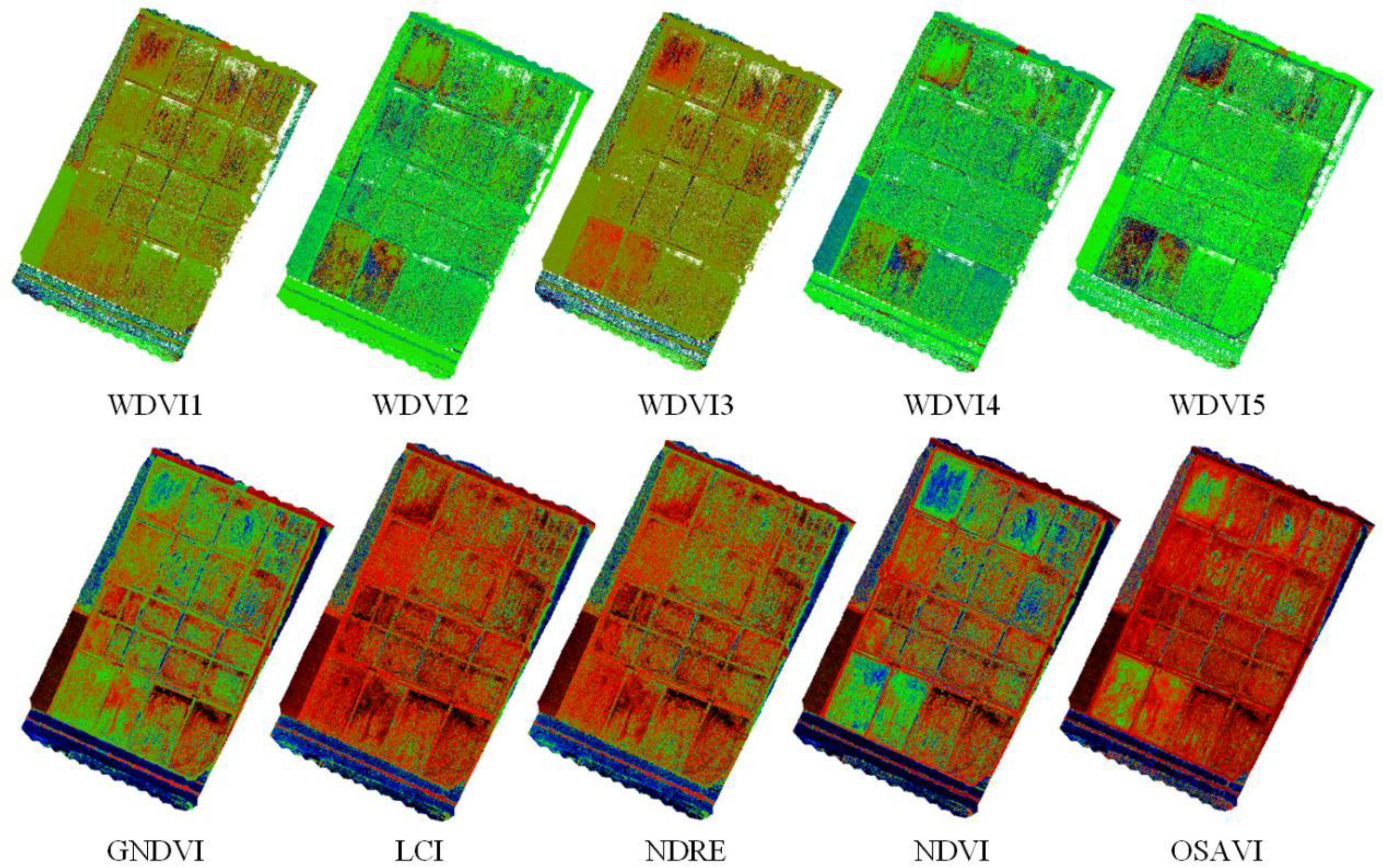
Results of weed identification with different vegetation indices.

The WDVI construction method was used and in this study five weed-sensitive indices were selected (Wan et al., 2020; Xia et al., 2021). Five traditional vegetation indices such as GNDVI(Green Normalized Difference Vegetation Index), NDVI(Normalized Difference Vegetation Index), LCI(Leaf Chlorophyll Index), NDRE(Normalized Differential Red Edge vegetation inde), and OSAVI(Optimized Soil Adjusted Vegetation Index) were selected for comparison, and a total of ten vegetation indices were used to generate pseudo-color maps for the identification of the rice weed vegetation index, and the results are shown in **Figure 3**.

It can be seen from **Figure 3** that different vegetation indices have different sensitivities to weeds in rice fields, and some fields have water cotton in them, but water cotton is different from weeds and requires different agents, so water cotton cannot be considered as a weed. From the effect of weed identification by different vegetation indices, the best result was obtained by using WDVI_NIR_.


(3) $$WDVI_{NIR}=\log_{\frac{\text{G}}{\text{NIR}}}\frac{RE}{NIR}$$

**Figure 4:**
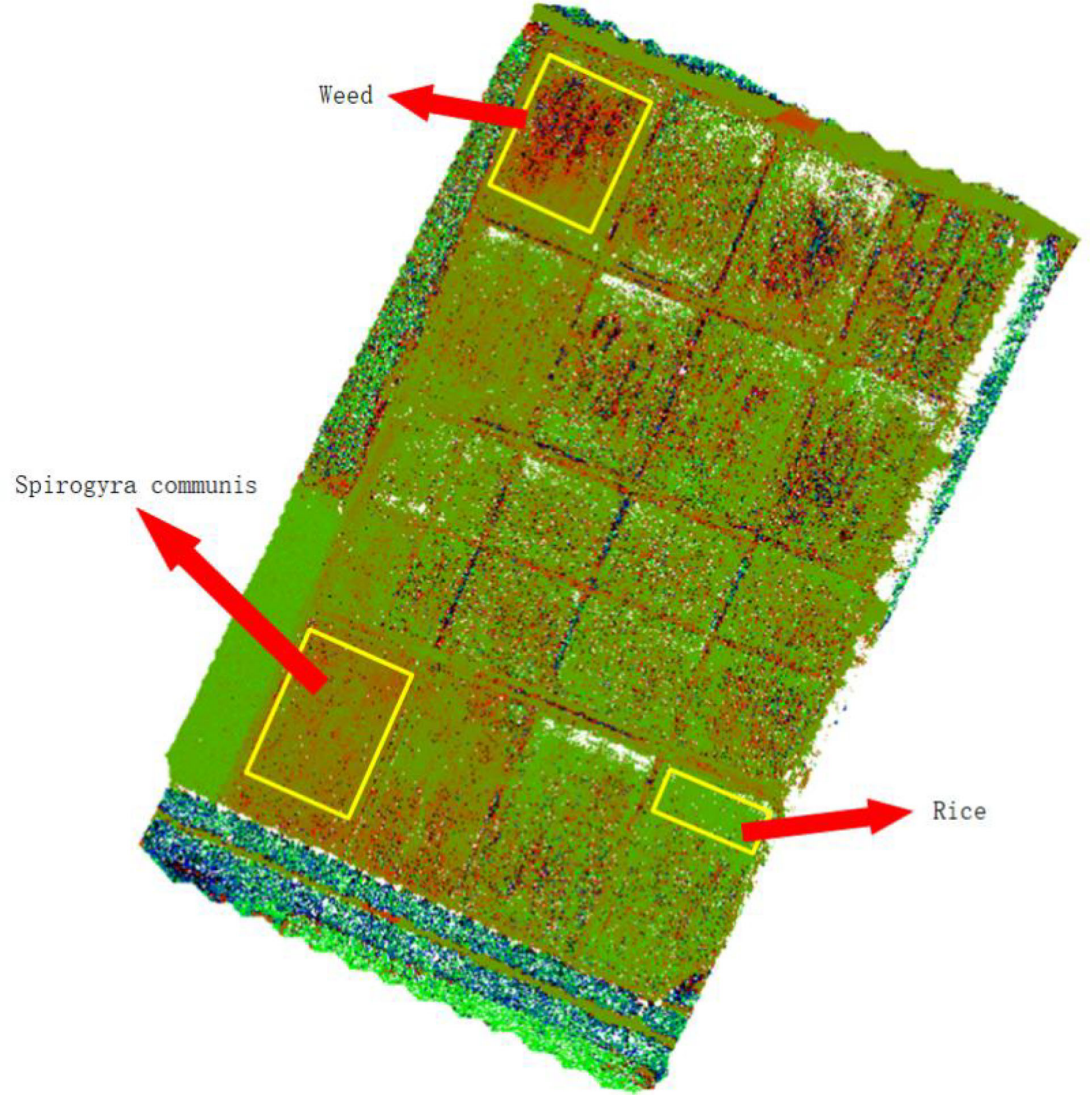
Results of *WDVI_NIR_* vegetation index.

In *WDVI*_*NIR*_ , NIR is the near-infrared wavelength reflectance of the multispectral UAV, G is the green wavelength reflectance, and RE is the red edge. *WDVI*_*NIR*_ can distinguish weeds from rice and spirogyra communis more clearly (**Figure 4**).

### 3.2 Results of rice weed classification based on density splitting

**Figure 5:**
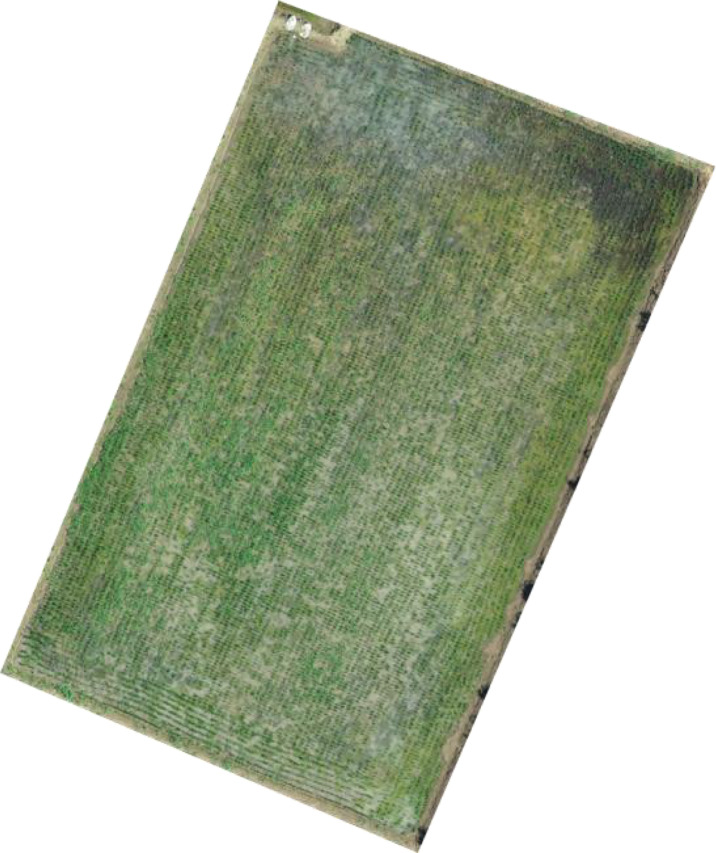
Visible image of field No. 9.

**Figure 6:**
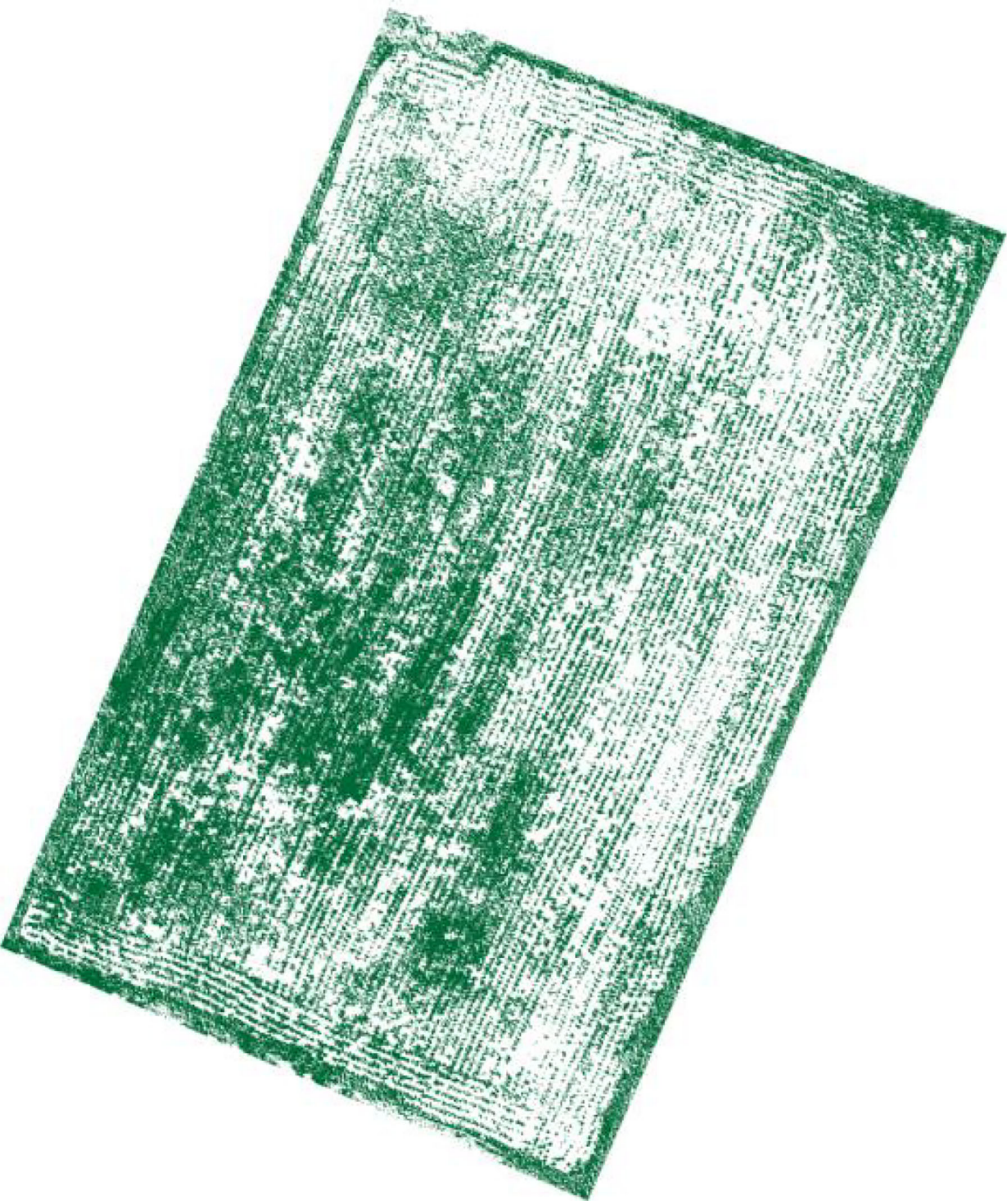
Density segmentation results.

Since the test area was large, the manual visual interpretation workload would be very large if the entire area were analyzed, so field 9 at **Figure 1**, where the number of weeds was at a medium level, was selected for analysis, and the visible light from the UAV in field 9 is shown in **Figure 5**. Using the manual visual interpretation method, the density segmentation threshold was determined using the criterion of covering all weeds. The results show that the density segmentation results can cover all weeds when the threshold values are 0 and 5. The results of the density segmentation are shown in **Figure 6**.

**Figure 7:**
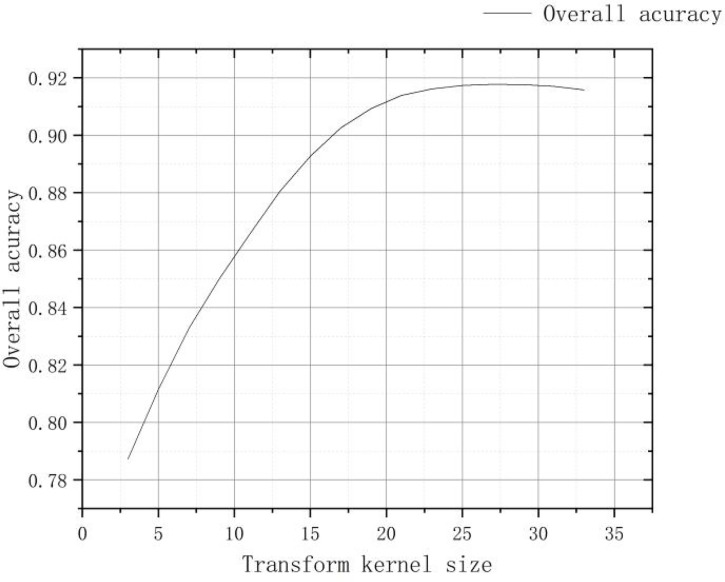
Confusion matrix verification accuracy curve after removing small patches.

**Figure 8:**
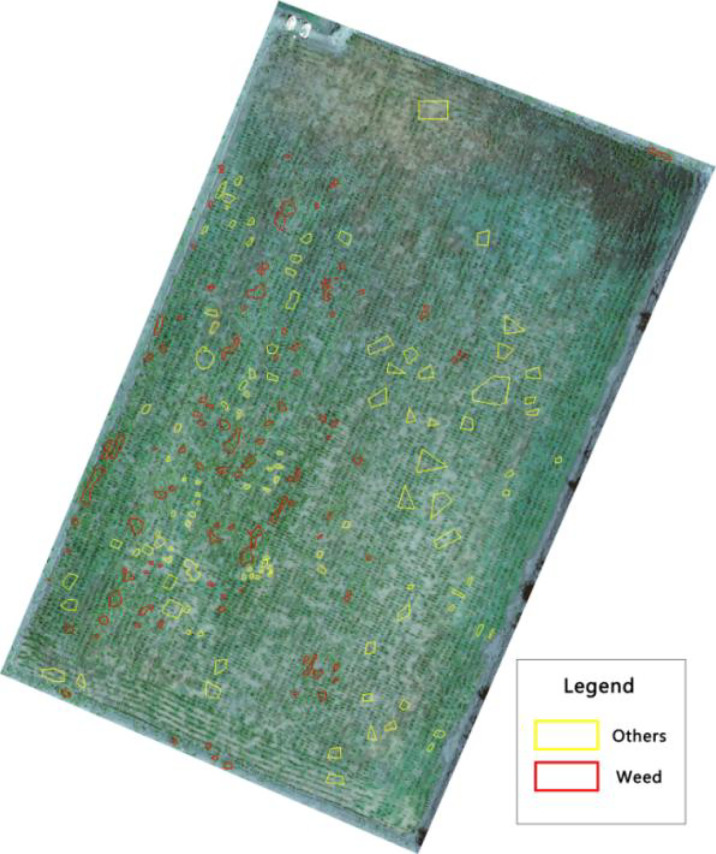
Manual visual interpretation vector map.

**Figure 9:**
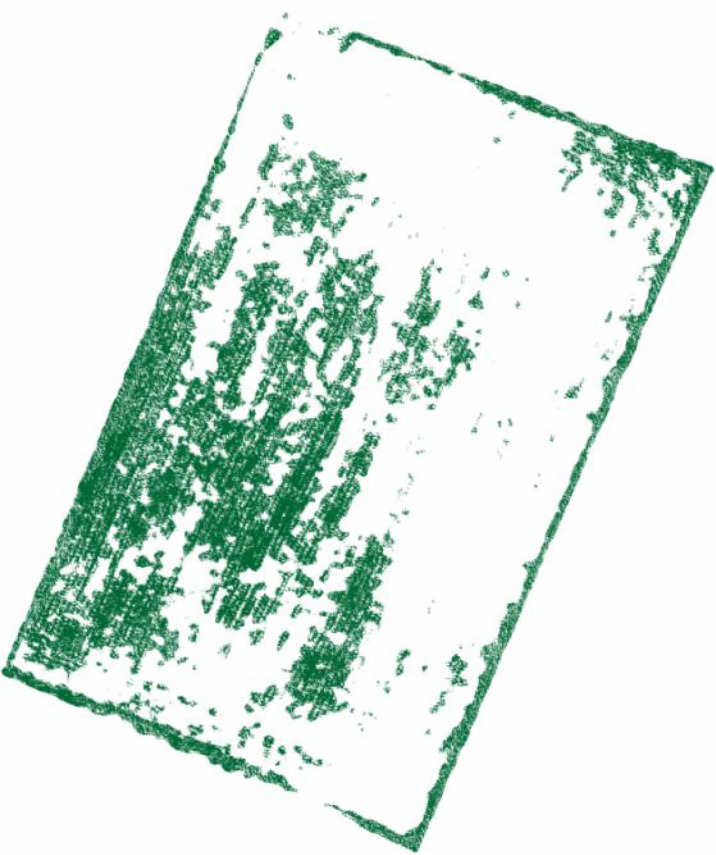
Results after removal of small plaques.

After density segmentation, the results were analyzed by removing small patches operation, using majority analysis method to remove small patches, and setting the transform kernel size as 3, 5, 7, 9, 11, 13, 15, 17, 19, 21, 23, 25, 27, 29, 31, 33, respectively. The results of the analysis are verified using the confusion matrix for accuracy, and the overall accuracy verification curve is shown in **Figure 7**, and the manual visual interpretation vector diagram used to verify the accuracy is shown in **Figure 8**. The verification results show that the highest accuracy of the confusion matrix verification is achieved when the size of the transformation kernel is set to 27, i.e., the best effect of removing small patches. The results after removing the small patches are shown in **Figure 9**.

**Figure 10:**
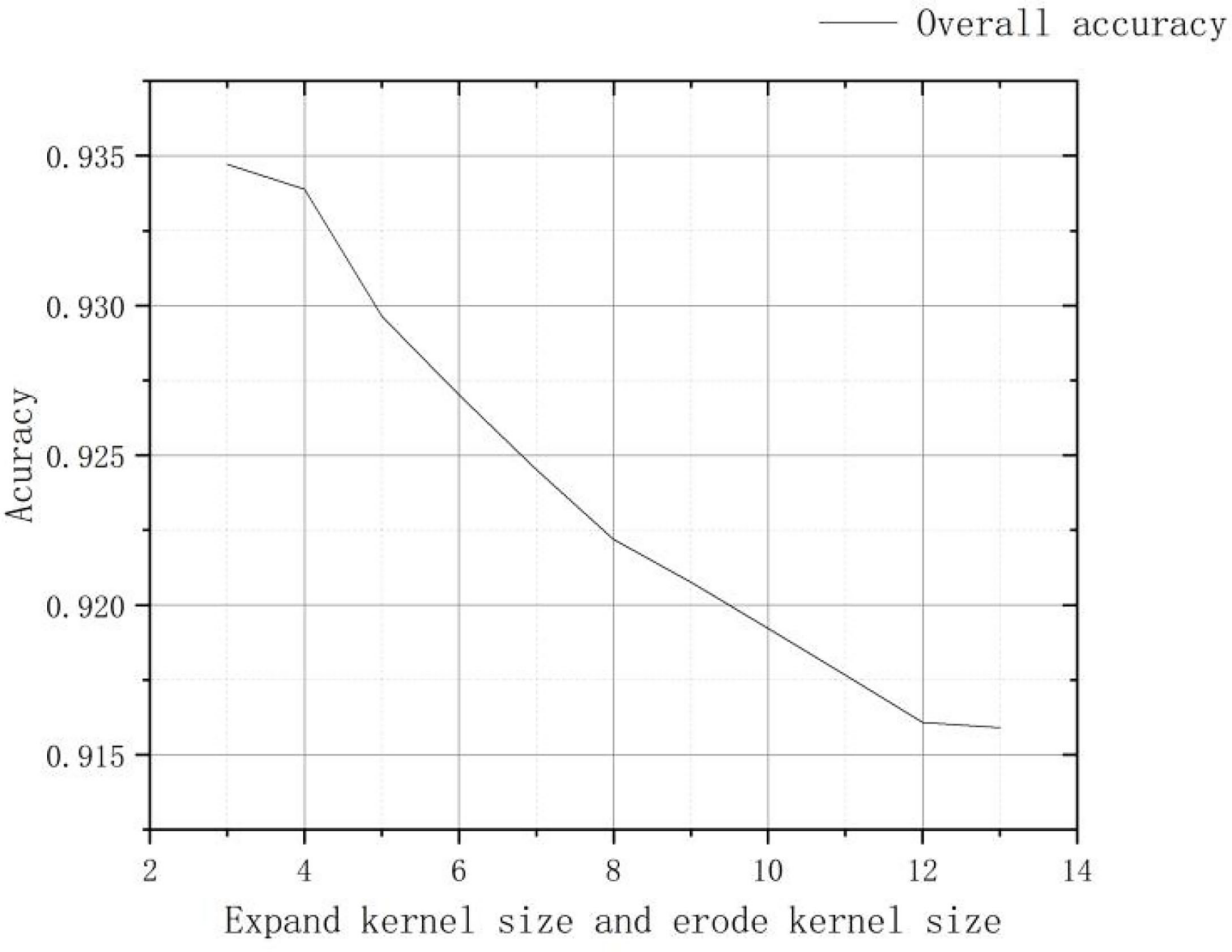
Confusion matrix verification accuracy curve after clustering process.

**Figure 11:**
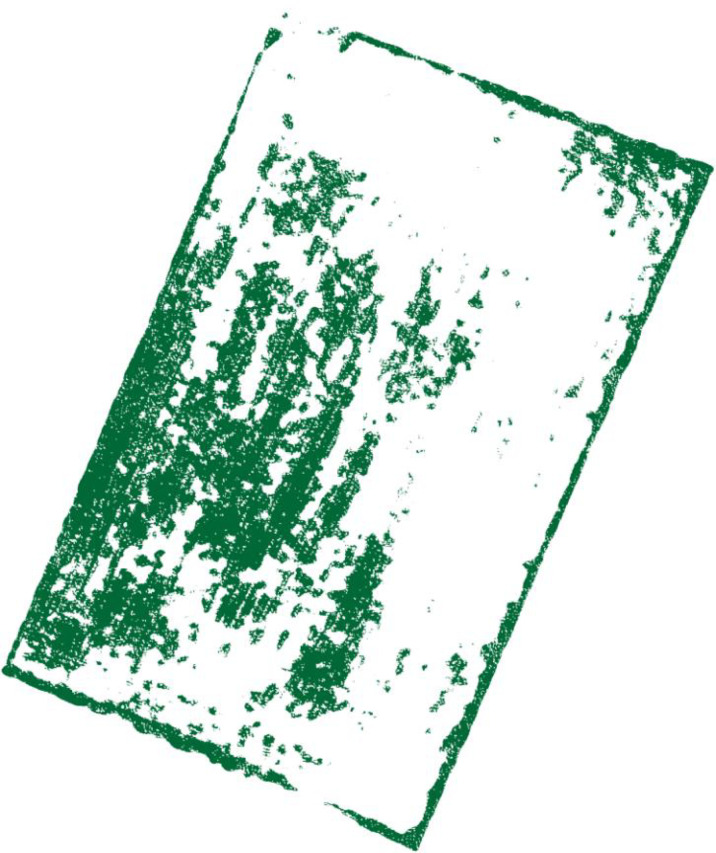
The result after clustering process.

The images processed by density segmentation and removal of small patches lack spatial continuity, which is not conducive to raster resampling operations during the production of UAV prescription maps. Therefore, the Clump Clustering algorithm is used for smoothing. The expansion kernel size and erosion kernel size are set to 3, 4, 5, 6, 7, 8, 9, respectively, and the kernel values are all 1 for cluster processing. The processed results are verified with precision using a confusion matrix, and the overall accuracy verification curves are shown in **Figure 10**. The validation results show that the overall accuracy of the confusion matrix is the highest when the expansion kernel size and the erosion kernel size are set to 3. The results after the clustering process are shown in **Figure 11**.

### 3.3 Weed UAV precision operation prescription map generation

**Figure 12:**
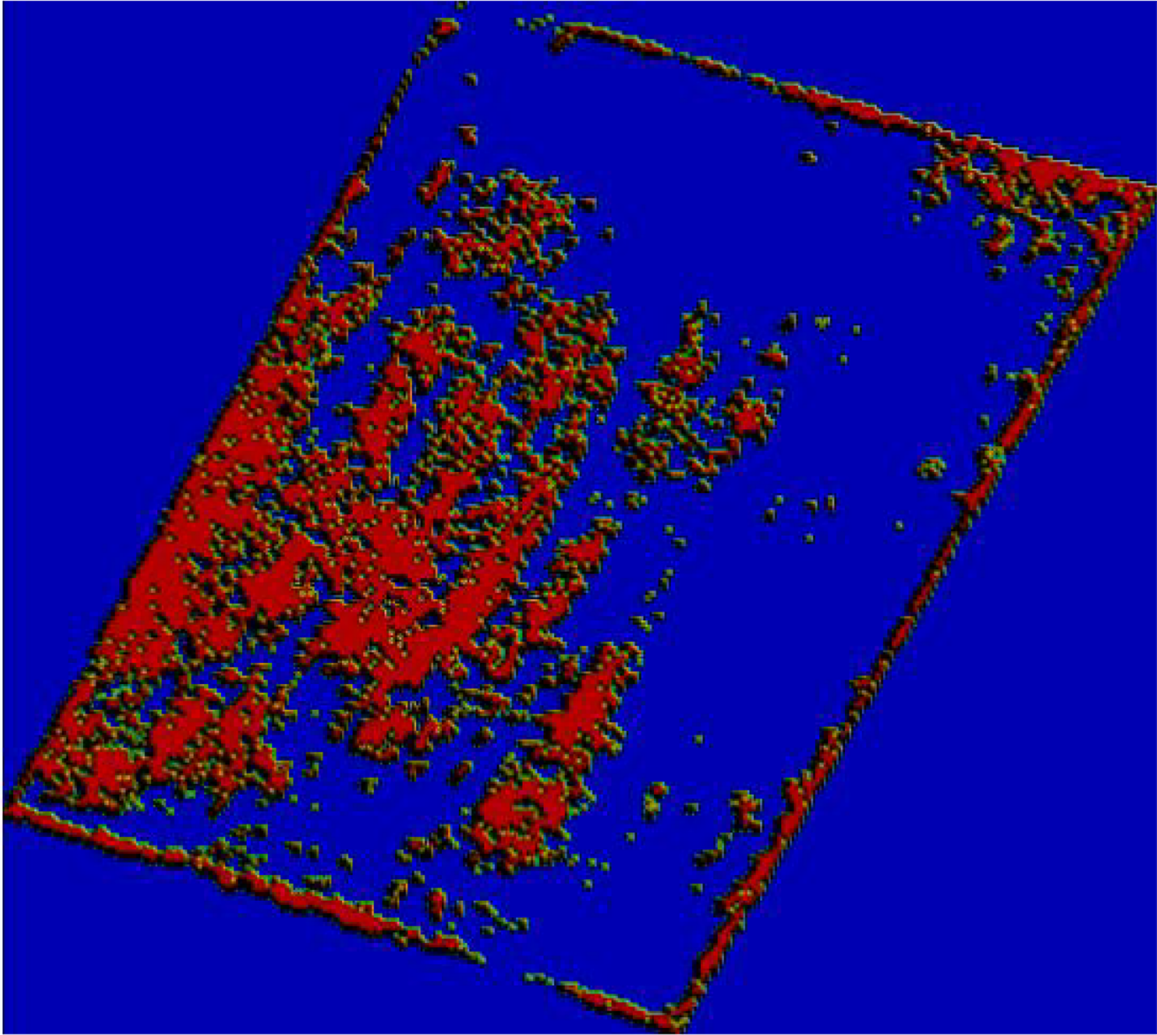
Before raster resampling.

**Figure 13:**
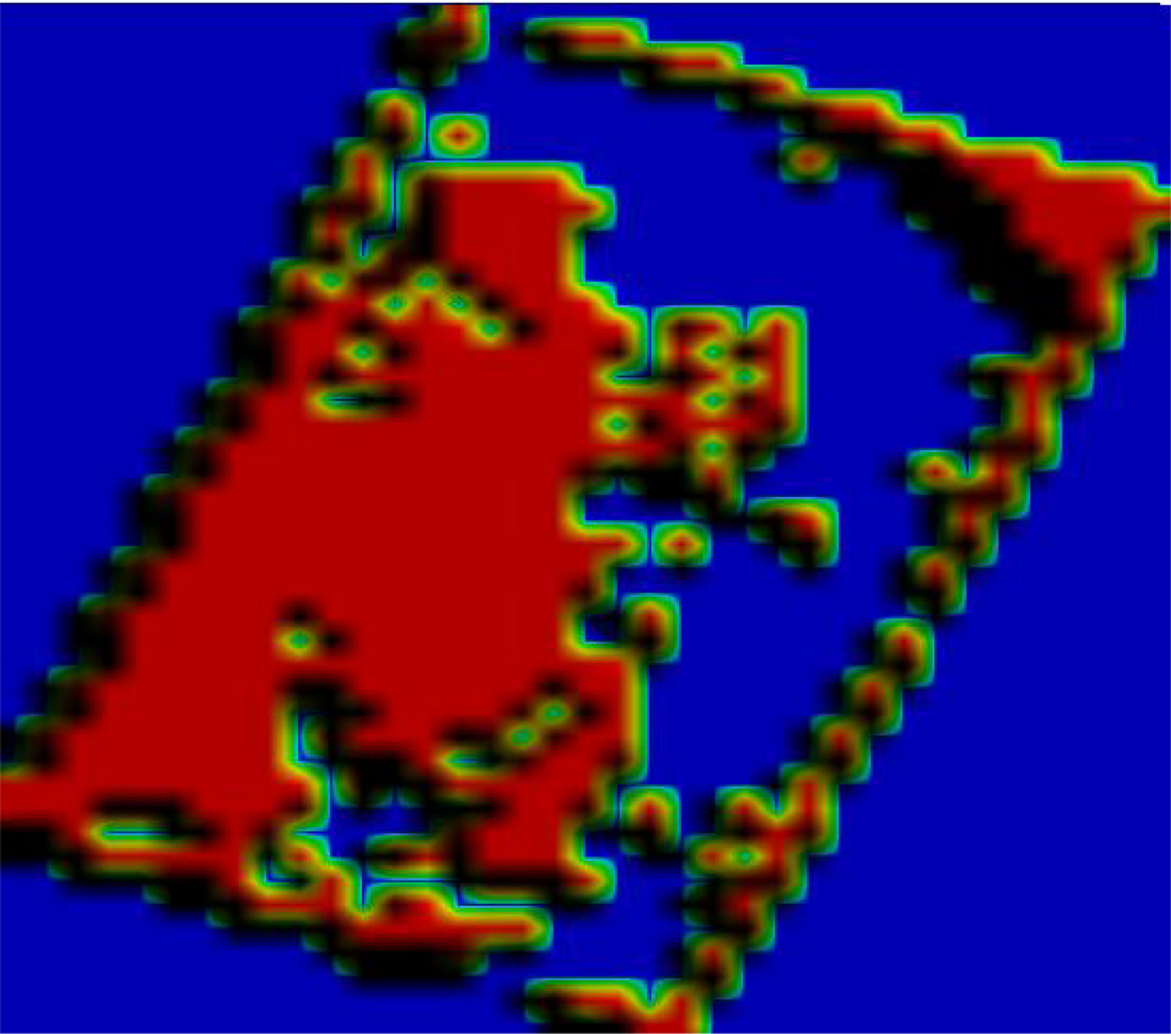
After raster resampling.

The UAV application operation must consider parameters such as flight speed and spray width of the plant protection UAV, and the prescription map must be raster data during the operation. Therefore, this study converts the vector data of weed identification results into raster data and resamples the raster data to the appropriate size. Take DJI plant protection drone T30 as an example, DJI T30 plant protection drone can operate 240 mu of fields per hour, the maximum operating flight speed is 7m/s, the volume of the operating tank is 30L, the number of nozzles is 16, the maximum effective spraying width is 4-9m, and the size of the prescription map grid required for operation is 1m*1m.Therefore, the raster data identified in this study are resampled to 1m*1m, and the raster data before resampling is shown in **Figure 12**, and the raster data after resampling are shown in **Figure 13**.

## 4 Discussion

Using UAV remote sensing technology to monitor weeds in rice fields and generate prescription maps to provide a decision basis for accurate herbicide application by plant protection machinery is one of the important methods to guide accurate rice weeding and is also a research focus of precision agriculture. We established a new weed-sensitive vegetation index using a low-cost UAV multispectral remote sensing platform, then used image recognition to accurately identify rice weeds and combined with GIS information to generate a prescription map for precise operation of agricultural drones for weeds in rice fields. The main idea of vegetation index construction in this study is to use mathematical transformation method to combine multispectral bands into a new vegetation index, and after RE and G are compared with NIR respectively, it is found that the ratio results have better sensitivity with weeds. The proposed WDVI vegetation index may also have decreased recognition accuracy and lack of generalizability when used in other field data sets. The reason for this may be that the vegetation index was constructed using data statistics and the mathematical mapping relationship between sensitive bands and weeds was not explored in the agronomic mechanism; the influence of different regions and varieties on the change in rice weeds was not considered in the research process (Yu et al., 2021). However, because the calculation of the vegetation index is simple and easy to realize the development and integration of detection devices, the method of rice weed identification based on the vegetation index still has considerable research value (Xia et al., 2022). The above problems should be explored and studied more deeply in future research experiments.

In this study, the accuracy of weed recognition in rice field was 93.47%. Compared with other scholars (Lan et al., 2021), it was found that the accuracy of weed recognition was comparable. However, compared with deep learning, spectral recognition of weeds has higher efficiency, saves time and requires less computing power, so it has more advantages.

In this study, we used manual labeling to tag multispectral remote sensing images from UAVs at pixel level for weed model training and accuracy verification. However, the manual labeling process is inefficient and time consuming. Manual tagging will affect the process of model development if remote sensing data increases substantially (Tobajas et al., 2020; Amziane et al., 2021). Therefore, in future research, it is necessary to introduce semi-supervised or weakly supervised analysis methods to reduce the workload of manual labeling. At the same time, remote sensing images are collected by a UAV, and a server is used offline to identify weeds and generate application prescription maps. In this mode of operation, data collection and data analysis are separated, and the best time for weed control is easily missed for weeds in larger rice production fields. Due to the current rapid development of the computing performance of embedded chips (Yang et al., 2022), which makes the real-time acquisition and analysis of UAV multispectral images possible, if the embedded chips can be deployed on UAVs and the analysis models on servers can be migrated to UAVs to realize the real-time processing of weed identification, the interval between data acquisition and data analysis can be effectively broken, and the process integration of UAV identification of weeds in fields can be realized, which will greatly enhance the application scope of remote sensing identification of weeds by UAV.

### 4.1 Conclusion

In this study, we created a weed identification index based on multispectral UAV images and constructed the *WDVI_NIR_* vegetation index from the reflectance of three bands, RE, G, and NIR. *WDVI*_*NIR*_ was compared with five traditional vegetation indices, NDVI, LCI, NDRE, and OSAVI, and the results showed that *WDVI*_*NIR*_ was the most effective for weed identification and could clearly distinguish weeds from rice, water cotton, and soil.

In this study, a weed identification method based on *WDVI*_*NIR*_ was constructed, and the weed index identification results were subjected to small patch removal and clustering processing operations to output weed identification vector results. The weed identification vector results were verified by using the confusion matrix accuracy verification method, and the results showed that the weed identification accuracy could reach 93.47%, and the Kappa coefficient was 0.859. Moreover, this study integrates the parameters of plant protection UAV operation and takes DJI UAV as an example to convert the weed recognition vector results into raster data with raster size of 1m*1m to make a UAV application prescription map for field application, which provides a new method for weed recognition in rice fields.

## Data availability statement

The raw data supporting the conclusions of this article will be made available by the authors, without undue reservation.

## Author contributions

FY and ZJ conceived and designed the experiments. SG, ZG, and HZ performed the experiments. FY analyzed the data. ZJ and SG wrote the original manuscript. FY, ZJ, TX, and CC reviewed and edited the original manuscript. All authors contributed to the article and approved the submitted version.

## Funding

This work was supported by the key research project of the Liaoning Provincial Department of Education (No. LSNZD202005).

## Conflict of interest

The authors declare that the research was conducted in the absence of any commercial or financial relationships that could be construed as a potential conflict of interest.

## Publisher’s note

All claims expressed in this article are solely those of the authors and do not necessarily represent those of their affiliated organizations, or those of the publisher, the editors and the reviewers. Any product that may be evaluated in this article, or claim that may be made by its manufacturer, is not guaranteed or endorsed by the publisher.

## References

[B1] Amziane A. Losson O. Mathon B. Dumenil A. Macaire L. (2021). Reflectance estimation from multispectral linescan acquisitions under varying illumination-application to outdoor weed identification. Sensors (Basel Switzerland) 21 (11), 3601. doi: 10.3390/S21113601 34064243 PMC8196826

[B2] Barrero O. Perdomo S. A. (2018). RGB And multispectral UAV image fusion for gramineae weed detection in rice fields. Precis. Agric. 19 (5), 809–822. doi: 10.1007/s11119-017-9558-x

[B3] Bouman B. A. M. Uenk D. Haverkort A. J. (1992). The estimation of ground cover of potato by reflectance measurements. Potato Res. 1 (4), 249–262. doi: 10.1007/BF02357604

[B4] Carroll J. (2020). Aerial imaging aids precision agriculture. Vision Syst. Design 1.

[B5] Clevers J. G. P. W. Verhoef W. (1993). LAI estimation by means of the WDVI: A sensitivity analysis with a combined PROSPECT-SAIL model. Remote Sens. Rev. 7 (1), 43–64. doi: 10.1080/02757259309532165

[B6] De Simone L. Ouellette W. Gennari P. (2022). Operational use of EO data for national land cover official statistics in Lesotho. Remote Sens. 14 (14), 3294. doi: 10.3390/RS14143294

[B7] Druskin V. Mamonov A. V. Zaslavsky M. (2021). Distance preserving model order reduction of graph-laplacians and cluster analysis. J. Sci. Computing 90(1), 1–30. doi: 10.1007/S10915-021-01660-3

[B8] Duarte L. Teodoro A. C. Sousa Joaquim J. Pádua L. (2021). QVigourMap: A GIS open source application for the creation of canopy vigour maps. Agronomy 11 (5), 952. doi: 10.3390/AGRONOMY11050952

[B9] Eppinga M. B. Baudena M. Haber E. A. Rietjerk M. Wassec M. J. Santos M. J. (2020). Spatially explicit removal strategies increase the efficiency of invasive plant species control. Ecol. Appl. Publ. Ecol. Soc. America. 31 (3), e02257. doi: 10.1002/eap.2257 PMC8047905 33159346

[B10] Feng S. Xu T. Y. Yu F. H. Wang N. Y. (2018). Research of method for inverting nitrogen content in canopy leaves of japonica rice in northeastern China based on hyperspectral remote sensing of unmanned aerial vehicle. Spectrosc. Spectral Anal. 39 (10), 3281–3287. doi: 10.3964/j.issn.1000-0593(2019)10-3281-07

[B11] González M.E.P. Revilla J.I.G. (2020). A new environmental and spatial approach to the tiwanaku world heritage site (Bolivia) using remote sensing (UAV and satellite images). Geoarchaeology 35 (3), 416–429. doi: 10.1002/gea.21778

[B12] Hirohiko M. (2002). Development of identification methods and elucidation of emergence ecology on gramineous weeds of paddy fields in Japan. J. Weed Sci. Technol. 47, 175–184. doi: 10.3719/weed.47.175

[B13] Jin X. Bagavathiannan M. McCullough P. E. Chen Y. Yu J. (2022). A deep learning-based method for classification, detection, and localization of weeds in turfgrass. Pest Manage. Sci. 78 (11), 4809–4821. doi: 10.1002/PS.7102 35900854

[B14] Kawamura K. Asai H. Yasuda T. Soisouvanh P. Phongchanmixay S. (2021). Discriminating crops/weeds in an upland rice field from UAV images with the SLIC-RF algorithm. Plant Production Sci. 24 (2), 198–215. doi: 10.1080/1343943X.2020.1829490

[B15] Lambert J. P. T. Childs D. Freckleton R. P. (2019). Testing the ability of unmanned aerial systems and machine learning to map weeds at subfield scales: a test with the weed alopecurus myosuroides (Huds). Pest Manage. Sci. 75 (8), 2283–2294. doi: 10.1002/ps.5444 PMC6767585 30972939

[B16] Lan Y. B. Huang K. H. Yang C. Lei L. C. Ye J. H. Zhang J. L.. (2021). Real-time identification of rice weeds by UAV low-altitude remote sensing based on improved semantic segmentation model. Remote Sens. 13 (21), 4370. doi: 10.3390/rs13214370

[B17] Liu H. J. Lee S. H. Saunders C. (2014). Development of a machine vision system for weed detection during both of off-season and in-season in broadacre no-tillage cropping lands. Am. J. Agric. Biol. Sci. doi: 10.3844/ajabssp.2014.174.193

[B18] Liu H. Yu Y. H. (2013). Affine translation surfaces in euclidean 3-space. Proc. Japan Academy Ser. A Math. Sci. 89 (9), 111–113. doi: 10.3792/pjaa.89.111

[B19] Liu H. Y. Zhu H. C. Li Z. H. Yang G. J. (2020). Quantitative analysis and hyperspectral remote sensing of the nitrogen nutrition index in winter wheat.Int. J. Remote Sens. 41, 858–881. doi: 10.1080/01431161.2019.1650984

[B20] Luo H. W. He L. X. Du B. Pan S. G. Mo Z. W. Duan M. Y.. (2020). Biofortification with chelating selenium in fragrant rice: effects on photosynthetic rates, aroma, grain quality and yield formation. Field Crops Res. 255, 107909. doi: 10.1016/j.fcr.2020.107909

[B21] Lu J. Zhang L. (2020). Data mining technology of computer testing system for intelligent machining. Neural Computing Appl. 2020, 1–11. doi: 10.1007/s00521-020-05369-6

[B22] Maes W. H. Steppe K. (2019). Perspectives for remote sensing with unmanned aerial vehicles in precision agriculture. Trends Plant Sci. 24, 152–164. doi: 10.1016/j.tplants.2018.11.007 30558964

[B23] Matsunami M. Matsunami T. Kokubun M. (2009). Growth and yield of new rice for Africa (NERICAs) under different ecosystems and nitrogen levels. Plant Prod. Sci. 12, 381–389. doi: 10.1626/pps.12.381

[B24] Mohidem N. A. Che’Ya N. Juraimi A. Ilahi F. W. F. Roslim M. H. M. Sulaiman N.. (2021). How can unmanned aerial vehicles be used for detecting weeds in agricultural fields? Agriculture 11 (10), 1004. doi: 10.3390/AGRICULTURE11101004

[B25] Motavalli S. Hossienzadeh M. Derafshi K. Alijani M. A. (2012). Coastline change detection using remote sensing and GIS at TONEKABON coast area during 1984 and 2010, MAZANDARAN PROVINCE, IRAN. Life Sci. J. 9 (4), 4174–4181. doi: 10.7537/marslsj090412.622

[B26] Naji T. A.H. (2018). Study of vegetation cover distribution using DVI, PVI, WDVI indices with 2D-space plot. J. Physics: Conf. Ser. 1003 (1), 012083. doi: 10.1088/1742-6596/1003/1/012083

[B27] Nawaz M. Mehmood Z. Bilal M. Munshi A. M. Rashid M. Yousaf R. M.. (2021). Single and multiple regions duplication detections in digital images with applications in image forensic. J. Intelligent Fuzzy Syst. 40 (6), 10351–10371. doi: 10.3233/JIFS-191700

[B28] Niu K. Bai S. H. Zhou L. M. Zhao B. Liu L. J. Yuan Y. W.. (2021). Design and experimental research of variable formula fertilization control system based on prescription diagram. Appl. Sci. 12 (1), 325. doi: 10.3390/APP12010325

[B29] Otsu K. Pla M. Duane. A. Brotons L. (2019). Estimating the threshold of detection on tree crown defoliation using vegetation indices from UAS multispectral imagery. Drones 3 (4), 80. doi: 10.3390/drones3040080

[B30] Punithavathi R. Rani A.D. C. Sughashini K. R. Kurangi C. Nirmala M. Ahmed H. F. T. (2023). Computer vision and deep learning-enabled weed detection model for precision agriculture. Comput. Syst. Sci. AND Eng. 44 (3), 2759–2774. doi: 10.32604/CSSE.2023.027647

[B31] Qin J. Q. Impa S. M. Tang Q. Y. Yang S. H. Yang J. Tao Y. S.. (2013). Integrated nutrient, water and other agronomic options to enhance rice grain yield and n use efficiency in double-season rice crop. Field Crops Res. 148, 15–23. doi: 10.1016/j.fcr.2013.04.004

[B32] Setojima M. Akamatsu Y. Hirose Y. (1989). Updating of actual vegetation map by plural satellite data and output of the vector map. J. Remote Sens. Soc. Japan 9 (2), 189–203. doi: 10.11440/rssj1981.9.189

[B33] Siva Kumar A. P. Vasumathi D. Naik M. V. (2020). An improved intelligent approach to enhance the sentiment classifier for knowledge discovery using machine learning. Int. J. Sensors Wireless Commun. Control 10 (4), 582–593. doi: 10.2174/2210327910999200528114552

[B34] Souri A. H. Chance K. Sun K. Liu X. Johnson S. (2022). Dealing with spatial heterogeneity in pointwise-to-gridded- data comparisons. Atmospheric Measurement Techniques 15 (1), 41–59. doi: 10.5194/AMT-15-41-2022

[B35] Stroppiana D. Villa P. Sona G. Ronchetti G. Candiani G. Pepe M.. (2018). Early season weed mapping in rice crops using multi-spectral UAV data. Int. J. Remote Sens. 39 (15-16), 5432–5452. doi: 10.1080/01431161.2018.1441569

[B36] Sujaritha M. Annadurai S. Satheeshkumar. J. Mahesh L. (2017). Weed detecting robot in sugarcane fields using fuzzy real time classifier. Comput. Electron. Agric. 134, 160–171. doi: 10.1016/j.compag.2017.01.008

[B37] Su J. Y. Yi D. W. Coombes M. Liu C. J. Zhai X. J. McDonald-Maier K.. (2022). Spectral analysis and mapping of blackgrass weed by leveraging machine learning and UAV multispectral imagery. Comput. Electron. Agric. 192, 106621. doi: 10.1016/J.COMPAG.2021.106621

[B38] Tao T. Wei X. H. (2022). A hybrid CNN–SVM classifier for weed recognition in winter rape field. Plant Methods 18 (1), 1–12. doi: 10.1186/S13007-022-00869-Z 35279179 PMC8917754

[B39] Tobajas J. C. Gallart M. Casamada J. L. Gil E. (2020). On-farm evaluation of prescription map-based variable rate application of pesticides in vineyards. Agronomy 10 (1), 102. doi: 10.3390/agronomy10010102

[B40] Wan L. Cen H. Y. Zhu J. P. Zhang J. F. Zhu Y. M. Sun D. W.. (2020). Grain yield prediction of rice using multi-temporal UAV-based RGB andmultispectral images and model transfer – a case study of small farmlands inthe south of China. Agric. For. Meteorol. 291, 108096. doi: 10.1016/j.agrformet.2020.108096

[B41] Wang L. L. Lan Y. B. Zhang Y. L. Chen P. C. (2019). Applications and prospects of agricultural unmanned aerial vehicle obstacle avoidance technology in China. Sensors (Basel Switzerland) 19 (3), 642. doi: 10.3390/s19030642 30717488 PMC6387432

[B42] Wang Z. Y. Wang Z. H. Li H. Ni P. Liu J. (2021). A modified approach of extracting landfast ice edge based on sentinel-1A InSAR coherence image in the gulf of bothnia. J. Mar. Sci. Eng. 9 (10), 1076. doi: 10.3390/JMSE9101076

[B43] Wei L. L. Luo Y. S. Xu L. Zhang Q. Cai Q. B. Shen M. J. (2021). Deep convolutional neural network for rice density prescription map at ripening stage using unmanned aerial vehicle-based remotely sensed images. Remote Sens. 14 (1), 46. doi: 10.3390/RS14010046

[B44] Xia Y. Fang. J. Chao. P. Pan. Z. H. Shang J. S. (2021). Cost-effective and adaptive clustering algorithm for stream processing on cloud system. GeoInformatica. doi: 10.1007/S10707-021-00442-1

[B45] Xia F. Quan L. Lou Z. Sun D. Li H. Lv X. (2022). Identification and comprehensive evaluation of resistant weeds using unmanned aerial vehicle-based multispectral imagery. Front. Plant Sci. 13, 938604. doi: 10.3389/FPLS.2022.938604 35937335 PMC9346607

[B46] Yang M. Xu X. Li Z. Y. Song X. Y. Xue H. Y. (2022). Remote sensing prescription for rice nitrogen fertilizer recommendation based on improved NFOA model. Agronomy 12 (8), 1804. doi: 10.3390/AGRONOMY12081804

[B47] Yu S. Q. Liu J. X. Han Z. Li Y. Tang Y. D. Wu C. D. (2021). Representation learning based on autoencoder and deep adaptive clustering for image clustering. Math. Problems Eng. 2021, 3742536. doi: 10.1155/2021/3742536

[B48] Zhu J. Wang J. DiTommaso. A. Zhou W. J. (2020). Weed research status, challenges, and opportunities in China. Crop Prot. 134, 104449. doi: 10.1016/j.cropro.2018.02.001

